# Administration of Onasemnogene Abeparvovec in an Infant With Spinal Muscular Atrophy and PCR-Confirmed SARS-CoV-2 Infection

**DOI:** 10.7759/cureus.92754

**Published:** 2025-09-19

**Authors:** Ikushi Shimomura, Shinsuke Maruyama, Yuichi Kodama, Manaka Matsunaga, Yasuhiro Okamoto

**Affiliations:** 1 Department of Pediatrics, Graduate School of Medical and Dental Sciences, Kagoshima University, Kagoshima, JPN

**Keywords:** coronavirus disease 2019, gene therapy, onasemnogene abeparvovec, severe acute respiratory syndrome coronavirus 2, spinal muscular atrophy, timing of administration, viral infections

## Abstract

Onasemnogene abeparvovec (OA) is used to treat spinal muscular atrophy (SMA), and early treatment is critical. However, OA administration may trigger an immune response. To date, no studies have established the optimal timing for OA administration following SARS-CoV-2 infection. We report the case of an eight-month-old female infant presenting with delayed motor development who was diagnosed with SMA. OA therapy was initially prescribed but postponed because of a risk of COVID-19 exposure; the patient’s mother had recently developed COVID-19. Despite testing positive for SARS-CoV-2, the infant remained asymptomatic, and her viral load did not increase. OA was administered 19 days after the mother’s COVID-19 diagnosis. The patient did not develop COVID-19 and experienced no significant adverse effects. This case suggests that OA administration may be safe in asymptomatic SARS-CoV-2-positive patients with low viral loads, allowing timely therapy to preserve motor function. Further studies are needed to establish evidence-based guidelines for the optimal timing of OA administration.

## Introduction

Spinal muscular atrophy (SMA) is an autosomal recessive neuromuscular disease that affects motor neurons in the anterior horn of the spinal cord and is characterized by progressive flaccid muscle weakness and motor regression [1]. SMA results from a deficiency of survival motor neuron (SMN) protein, caused by biallelic deletions or pathogenic variants of the SMN1 gene [1].

Therapeutic options for SMA include nusinersen, risdiplam, and onasemnogene abeparvovec (OA), with earlier treatment associated with improved efficacy [1]. OA is an in vivo gene replacement therapy that uses adeno-associated virus serotype 9 to deliver the SMN1 gene into anterior horn cells, enabling production of full-length SMN protein.

OA administration may trigger an immune response, including fever, elevated aminotransferase levels, and thrombocytopenia. Therefore, patients should be screened for undetectable anti-AAV9 antibody titers and for underlying medical conditions that may increase the risks associated with AAV9 therapy. Prednisolone (PSL) must be coadministered with OA to mitigate immune-related adverse effects [2]. Infectious diseases related to immune status, including liver dysfunction, have been reported both before and after OA treatment [3]. Under SARS-CoV-2 infection, immunosuppression increases the risks of severe illness and viral reactivation. However, the optimal timing between infection diagnosis and OA initiation remains unclear.

To date, no reports have described OA administration in SARS-CoV-2-positive patients, based on a PubMed search. To the best of our knowledge, this is the first report describing OA administration following SARS-CoV-2 infection.

## Case presentation

An eight-month-old female infant, the third child of non-consanguineous parents, was referred for evaluation. The patient’s older sister (the first child) had been diagnosed with SMA type 2. The patient was born at 38 weeks of gestation, with a birth weight of 3,030 g, and had not been screened for SMA. There were no notable perinatal complications.

At eight months of age, the patient was unable to sit independently and was therefore evaluated at another hospital in Kagoshima City (day 0). SMA was suspected, genetic testing was performed, and the patient was referred to the Department of Pediatrics at Kagoshima University Hospital. On day 8, she was diagnosed with SMA due to the absence of exons 7 and 8 in *SMN1* and the presence of three copies of *SMN2*.

OA administration was initially scheduled for day 13; however, it was postponed because the patient’s mother tested positive for COVID-19 on day 12. On day 27, a SARS-CoV-2 PCR test yielded a cycle threshold (Ct) value of 32. The Ct value represents the number of cycles required for the PCR test to detect the virus, providing an estimate of viral load in the sample. At our hospital, a Ct value above 30 was considered indicative of a low viral load and was used as a criterion for lifting quarantine, resuming chemotherapy, and similar interventions. Because infection before or after OA treatment could lead to more severe complications, therapy was deferred due to the potential for COVID-19 progression.

On day 29, the patient remained asymptomatic, and repeat PCR testing revealed a Ct value of 29. At that point, she was considered to have a low viral load with asymptomatic COVID-19.

**Figure 1:**
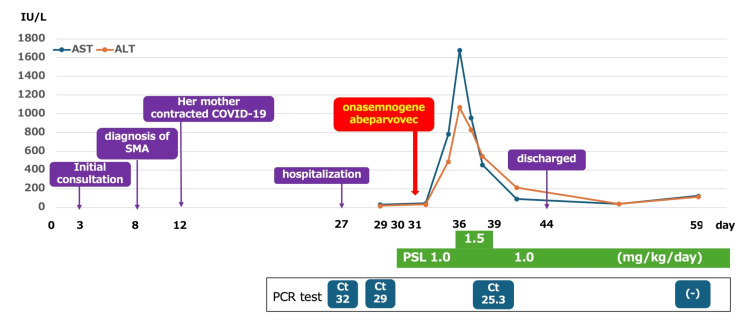
Summary of patient progress ALT, alanine aminotransferase; AST, aspartate aminotransferase; Ct, cycle threshold; PSL, prednisolone; SMA, spinal muscular atrophy; (-), PCR negative

Oral PSL at 1.0 mg/kg/day was initiated on day 30, and OA (1.1 × 10¹⁴ vg/kg) was administered on day 31. On day 34, the patient developed a fever of 38 °C, which resolved spontaneously. On day 36, laboratory testing revealed aspartate aminotransferase (AST) and alanine aminotransferase (ALT) levels exceeding 1,000 IU/L, accompanied by mild vomiting. The PSL dose was increased to 1.5 mg/kg/day. Liver enzyme levels peaked at AST 1,627 IU/L on day 36 and ALT 1,067 IU/L on day 39, after which the PSL dose was reduced to 1.0 mg/kg/day. PCR testing for SARS-CoV-2 at that time revealed a Ct value of 25.3 (Figure 1).

The patient remained in good general condition and was discharged on day 44. On day 59, PCR testing for SARS-CoV-2 was negative. PSL therapy was continued until day 113 due to prolonged elevation of aminotransferase levels, after which the dose was tapered every two weeks and discontinued on day 143. At one year and one month of age, the patient’s Children’s Hospital of Philadelphia Infant Test of Neuromuscular Disorders score was 62, indicating improved motor function.

## Discussion

Several side effects have been associated with OA administration, the most common being elevated aminotransferase levels, possibly related to hepatic uptake of AAV9. In severe cases, CD8+ cell infiltration and liver fibrosis have been reported. PSL must be coadministered with OA to mitigate immune-related adverse effects, including liver dysfunction [2].

Infectious diseases are a known risk factor. Experts recommend waiting at least two weeks after resolution of an active infection before initiating OA therapy, as infections occurring before or after infusion may lead to more severe complications [3]. One safety report described OA administration 9 days after resolution of acute disease symptoms [4]. In contrast, Sakemi et al. [5] reported a case of acute liver failure triggered by a respiratory virus infection two months after OA administration, and Feldman et al. [6] described norovirus-associated acute liver failure seven weeks post-OA. To our knowledge, no detailed reports have addressed SARS-CoV-2 infection before or after OA administration. A real-world pharmacovigilance study reported 44 cases of COVID-19 following OA administration, though details remain unclear [7].

In the present case, OA was initially withheld due to potential risks associated with concurrent viral infection, including the possibility of viral reactivation leading to COVID-19 or exacerbating drug-related side effects. On the other hand, data indicate that OA efficacy is highly dependent on early administration [8]; thus, timely therapy is critical to achieving maximal therapeutic benefit. COVID-19 typically manifests within three to seven days following an initial positive PCR result [9], after which viral load does not usually continue to rise. Accordingly, we assessed this patient as having an asymptomatic, non-active SARS-CoV-2 infection and proceeded with OA administration without delay. Recurrent PCR testing showed a Ct value around 30, suggesting a low likelihood of false-positive results, and the findings may reflect residual viral RNA. Ultimately, each case requires an individualized risk-benefit assessment and multidisciplinary decision-making that includes parental input.

Although the patient developed a fever and elevated aminotransferase levels, these were mild and within the expected range based on prior reports. We determined that the timing of these events indicated drug-related side effects. The favorable clinical course may have been due to the absence of overt COVID-19 symptoms despite PCR positivity. We believe that the timing of OA administration and corticosteroid therapy was appropriate, as no serious adverse effects or infections occurred. While determining the optimal timing of OA initiation following infection remains complex, this case demonstrates for the first time that OA can be safely administered in asymptomatic SARS-CoV-2-positive patients.

This case report has several limitations. First, as a single case, it cannot definitively establish the safety of OA administration in SARS-CoV-2-positive patients. Second, long-term follow-up data are lacking. Third, viral characteristics were not fully characterized.

## Conclusions

Although infection is a critical risk factor, OA can be administered when asymptomatic SARS-CoV-2 positivity presents a low risk of disease progression, provided a careful risk-benefit assessment is performed. While this case offers valuable insights into the management of SMA and the use of gene therapy in patients with concurrent viral infections, it does not establish clinical recommendations. Further case collection is needed to determine best practices for timing OA administration following various infections.
